# Meta-analysis of Clinical Microbiome Studies in Urolithiasis Reveal Age, Stone Composition, and Study Location as the Predominant Factors in Urolithiasis-Associated Microbiome Composition

**DOI:** 10.1128/mBio.02007-21

**Published:** 2021-08-10

**Authors:** Naveen Kachroo, Dirk Lange, Kristina L. Penniston, Joshua Stern, Gregory Tasian, Petar Bajic, Alan J. Wolfe, Mangesh Suryavanshi, Andrea Ticinesi, Tiziana Meschi, Manoj Monga, Aaron W. Miller

**Affiliations:** a Glickman Urological and Kidney Institute, Cleveland Clinic, Cleveland, Ohio, USA; b The Stone Centre at VGH, Department of Urologic Sciences, University of British Colombia, Vancouver, British Columbia, Canada; c Department of Urology, University of Wisconsin—Madison School of Medicine and Public Health, Madison, Wisconsin, USA; d Department of Urology, Intermountain Healthcare, Salt Lake City, Utah, USA; e Division of Urology, The Children’s Hospital of Philadelphia and Departments of Surgery and Biostatistics, Epidemiology and Informatics, University of Pennsylvania, Philadelphia, Pennsylvania, USA; f Department of Microbiology and Immunology, Loyola University—Chicago, Maywood, Illinois, USA; g Yenepoya Research Centre, Yenepoya University, Mangalore, India; h Geriatric-Rehabilitation Department, Azienda Ospedaliero-Universitaria di Parma, Parma, Italy; i Department of Medicine and Surgery, University of Parma, Parma, Italy; j Department of Urology, University of California—San Diego School of Medicine, La Jolla, California, USA; k Department of Cardiovascular and Metabolic Sciences, Cleveland Clinic, Cleveland, Ohio, USA; Louis Stokes Veterans Affairs Medical Center

**Keywords:** urolithiasis, metagenomics, microbiome, meta-analysis, kidney stone, clinical, metagenome

## Abstract

To determine whether functionally relevant questions associated with the urinary or gut microbiome and urinary stone disease (USD) can be answered from metagenome-wide association studies (MWAS), we performed the most comprehensive meta-analysis of published clinical MWAS in USD to date, using publicly available data published prior to April 2021. Six relevant studies met inclusion criteria. For alpha-diversity, significant differences were noted between USD status, stone composition, sample type, study location, age, diet, and sex. For beta-diversity, significant differences were noted by USD status, stone composition, sample type, study location, antibiotic use (30 days and 12 months before sampling), sex, hypertension, water intake, body habitus, and age. *Prevotella* and *Lactobacillus* in the gut and urinary tract, respectively, were associated with healthy individuals, while *Enterobacteriaceae* was associated with USD in the urine and stones. Paradoxically, other *Prevotella* strains were also strongly associated with USD in the gut microbiome. When data were analyzed together, USD status, stone composition, age group, and study location were the predominant factors associated with microbiome composition. Meta-analysis showed significant microbiome differences based on USD status, stone composition, age group or study location. However, analyses were limited by a lack of public data from published studies, metadata collected, and differing study protocols. Results highlight the need for field-specific standardization of experimental protocols in terms of sample collection procedures and the anatomical niches to assess, as well as in defining clinically relevant metadata and subphenotypes such as stone composition.

## INTRODUCTION

Urinary stone disease (USD) has increased in prevalence 4-fold in the last 50 years and has seen an epidemiological shift to earlier disease onset (1), with a recurrence rate of 30% within 10 years after an initial stone episode (2). Understanding the causal relationships driving these changes are crucial to identifying potentially modifiable risk factors and therapeutic strategies. USD, like many chronic inflammatory conditions, is considered a multifactorial disease with numerous stone phenotypes, environmental and metabolic risk factors, such as age, host genetics, diet, sex, and medication use (3). The microbiota is strongly associated with these risk factors, a finding that implicates environmentally driven changes within the microbiota as an important mediator in pathogenesis of USD. Prior work in the field has predominantly focused on standard culture or PCR-based methods (4), which are known to have significant limitations (5). Advancements in high-throughput and culture-independent techniques have allowed for a more in-depth exploration of the microbiota. Numerous culture-independent microbiota studies have been published since 2016 (5–16) attempting to address the question of whether the microbiota contributes to the onset of USD. While published studies comparing the microbiota of USD patients with that of healthy controls share some similarities in results, clear differences are apparent in terms of the metadata associations, along with the specific bacteria driving those associations. Clinical metagenome-wide association studies (MWAS) are relatively new. As such, the costs, access to sequencing technologies and bioinformatic expertise are often significant barriers for clinicians. Furthermore, questions remain about the reproducibility, applicability, and physiological relevance of these MWAS, particularly if variation in results are due to differences in the experimental design (i.e., sample collection, storage, DNA extraction, sequencing, or data analysis) or population characteristics (i.e., geography, ethnicity, disease subphenotypes, or some other regional factors).

An important consideration to address the above issues is the choice of taxonomic assignment for the sequencing data. Taxonomic assignment to operational taxonomic units (OTUs) or amplicon sequence variants (ASVs) are two different means to classify sequence reads and produce count tables prior to subsequent analyses. Traditionally, OTUs are assigned based either on sequence homology to a reference database (closed reference) or as a function of pairwise sequence homology (*de novo*). One caveat to OTU assignment is that sequencing errors can lead to misclassifications, chimeric sequences, and an inflation of the number of taxa defined (17). The more recent ASV classification strategy is a *de novo* process designed to overcome the issues with OTU assignment. This strategy assumes that biological variants are more repeatable than sequencing errors and thus limits the impact of sequencing errors on assignment. Classification by ASV is thought both to be more accurate than strategies that define OTUs and are consistently defined across independent data sets (18), thereby potentially making collaborative studies easier to conduct. However, few studies have compared the OTU and ASV classification strategies with real-world data (18).

To translate the results of MWAS studies into actionable interventions, there are several considerations regarding the interaction between the microbiome and chronic inflammatory diseases, such as USD. First, one must identify the optimal source of microbial activity that is most relevant to the pathogenesis of the disease. USD microbiome studies have predominantly focused on the gut microbiome, with few reports on the urinary tract or stone microbiome. Second, identification of specific taxa that drive dysbiosis and hence may influence the disease is necessary. Finally, we must understand how metadata (patient and environmental characteristics) affect the relationship between the disease in question and the microbiome. Of particular importance are specific disease subphenotypes that may arise from unique physiological origins. In USD, for instance, numerous types of stones can manifest that may result from metabolic disorders, dietary choices, infections, or genetic conditions (3). The data from MWAS, given the above considerations, can provide the foundation for rationally designed mechanistic studies to confirm or refute disease causality and thus lead to targeted interventions for primary and secondary prevention of diseases such as USD. Thus, the aim of this study was to perform the most comprehensive meta-analysis of all currently published clinical MWAS in USD to determine whether clinically relevant questions can be answered from the existing literature and whether the experimental design impacts the results of individual studies.

## RESULTS

### Microbiome meta-analysis.

In our microbiome meta-analysis, six relevant studies were eligible for inclusion with representative samples from the stool, urine, and stones, and locations that included USA (7, 16), Canada (8), India (10), China (9) and Italy (12) spanning 201 patient samples and 136 control samples (Table 1). There was no significant heterogeneity in alpha- and beta-diversity results between studies (*P *> 0.05).

**TABLE 1:** Clinical microbiome studies included in meta-analysis

Study (reference)	Location	Study cohort		Sample	Dataset	Platform
		USD	Controls			
Dornbier et al. (16)	Chicago, USA	71	0	Urine stone	16S rRNA	Illumina MiSeq
Zampini et al. (7)	Cleveland, USA	24	43	Urine stool stone	16S rRNA	Illumina MiSeq
Miller et al. (8)	Vancouver, Canada	17	17	Stool	16S rRNA	Illumina MiSeq
Tang et al. (9)	Nanning, China	13	13	Stool	16S rRNA	Illumina HiSeq
Suryavanshi et al. (10)	Sutarwadi, India	24	15	Stool	16S rRNA	Ion Torrent
Ticinesi et al. (12)	Parma, Italy	52	48	Stool	16S rRNA	Illumina MiSeq

**FIG 1:**
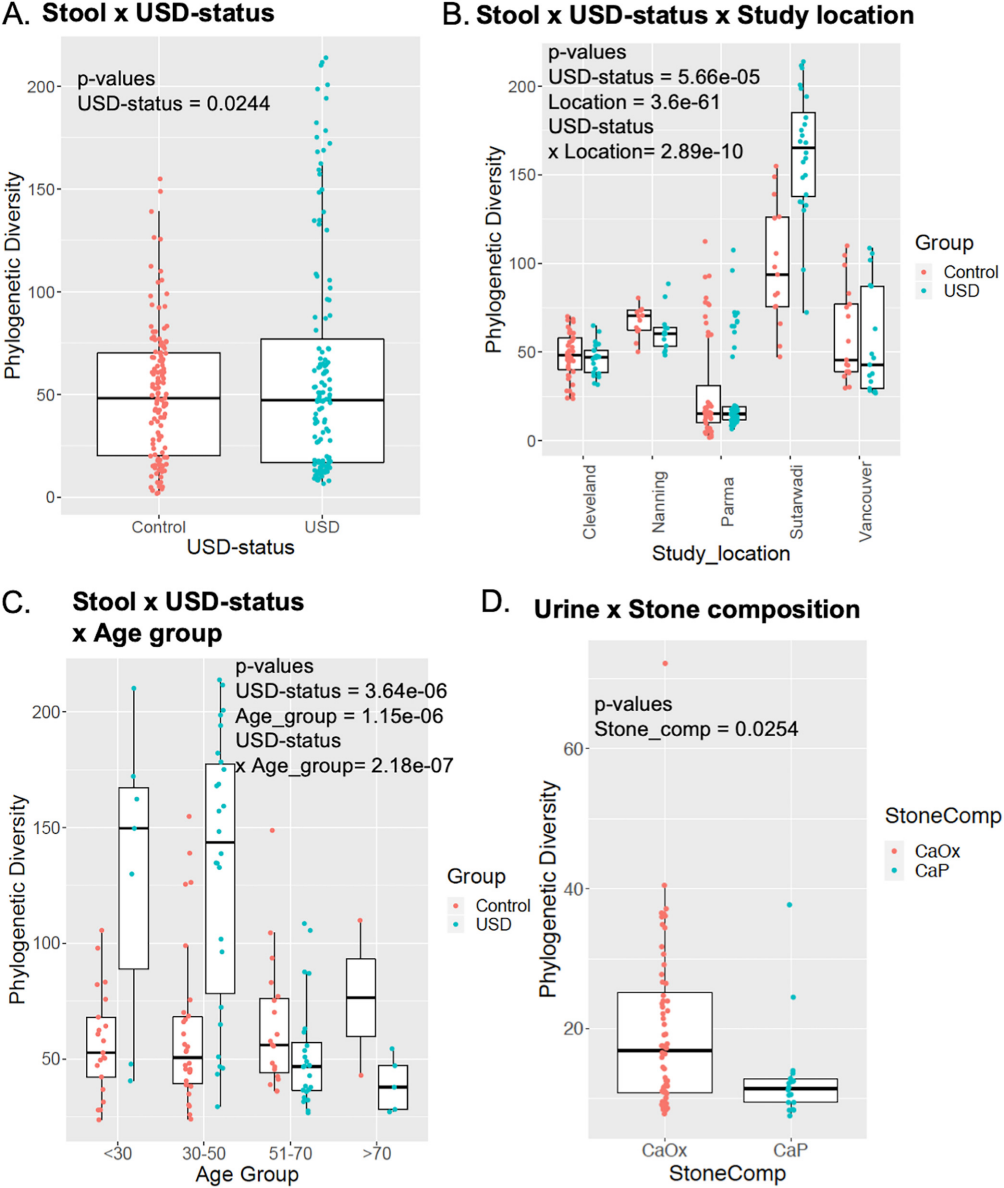
Significant alpha diversities from microbiome study meta-analysis with OTUs. (A) USD status for stool across all studies. (B) USD status in stool samples from different study locations: Cleveland (USA), Nanning (China), Vancouver (Canada) and Sutarwadi (India). (C) USD status and age-group for stool. Age groups include <30 years old, 30 to 50 years old, 51 to 70 years old, and >70 years old.

**FIG 2:**
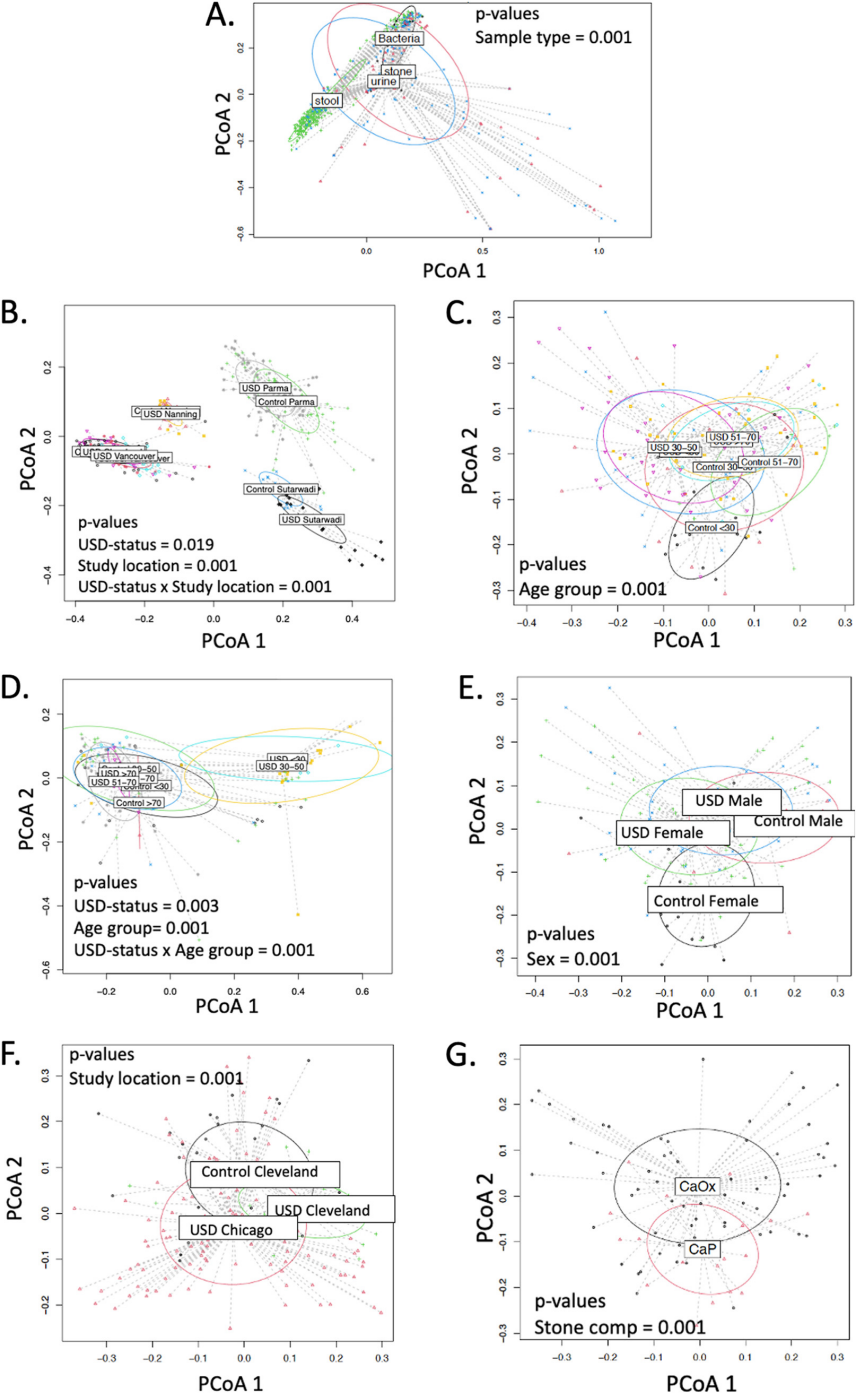
Significant beta diversities from microbiome study meta-analysis with OTUs. (A) Sample type comparison across all studies. (B) USD status in stool samples from different study locations: Cleveland (USA), Nanning (China), Vancouver (Canada), and Sutarwadi (India). (C) USD status and age group for stool. Age groups include <30 years old, 30 to 50 years old, 51 to 70 years old, and >70 years old. (D) Study locations for urine: Cleveland (USA) and Chicago (USA). (E) Age group for urine. (F) Sex for urine.

**FIG 3:**
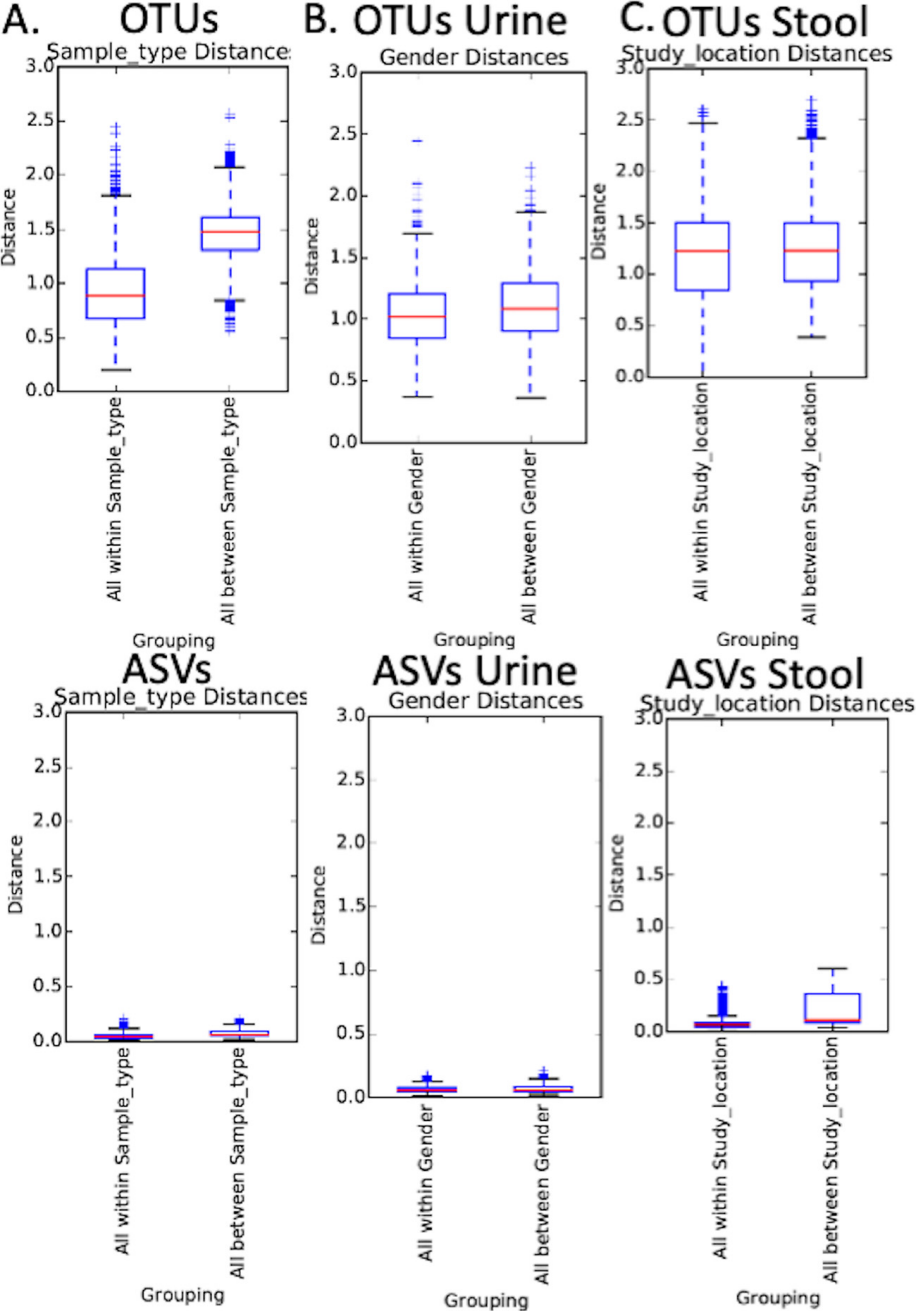
Discriminatory power of OTUs versus ASVs for physiologically distinct metrics. The discriminatory power of OTUs and ASVs was quantified using the within group and between group variance in beta-diversity as assessed through weighted UniFrac distances for three physiologically relevant metrics. (A) Sample type (stool, urine, and kidney stone). Only one study (7) included raw data and metadata for more than one sample type and is the only study included here. (B) Sex for the urinary microbiome. Only one study (7) included raw data and metadata for the urinary microbiome and is the only study included here. (C) Study location. All studies were included and the analysis was based on the stool microbiome only. *, False discovery rate corrected *P* values < 0.05.

When stratifying by USD status, there were significant differences in both alpha- and beta-diversity between studies that classified taxa using OTU. Further stratification by stone composition, age, and study location found significant differences when taxa were classified by either OTU or ASV (Fig. 1 and 2 and Table 2) (Table 2; see also Fig. S1 and S2 in the supplemental material). Associations between USD status and all other metadata were nonsignificant. For physiologically distinct phenotypes, such as sample type (stool, stone, and urine), urinary tract delineated by sex, and study location, OTU classification provided greater discriminatory power than ASV classification (Fig. 3).

**TABLE 2:** All significant microbiome results for alpha- and beta-diversity associations with clinical metadata^*a*^

Comparison and sample(s)	Metadata	Operational taxonomic units							Amplicon sequence variants						
		10	9	12	8	7	16	M-A	10	9	12	8	7	16	M-A
Alpha-diversity															
Stool	Age group	0.704	NA	NA	0.409	0.865	NA	**<0.001**	0.588	NA	NA	0.259	0.421	NA	0.844
Stool	Desserts	NA	NA	NA	NA	**0.046**	NA	NA	NA	NA	NA	NA	0.699	NA	NA
Stool	USD status × diet	0.467	NA	NA	NA	0.127	NA	0.841	0.319	NA	NA	NA	**0.004**	NA	0.744
Stool	USD status × meat	NA	NA	NA	NA	0.578	NA	NA	NA	NA	NA	NA	**0.005**	NA	NA
Stool	USD status	**<0.001**	0.848	0.837	0.404	0.606	NA	**0.024**	**<0.001**	0.237	0.103	0.32	0.084	NA	0.567
Stool	USD status × age group	0.504	NA	NA	0.148	0.795	NA	**<0.001**	0.996	NA	NA	**0.006**	0.166	NA	**0.014**
Stool	USD status × geographic location	NA	NA	NA	NA	NA	NA	**<0.001**	NA	NA	NA	NA	NA	NA	**0.054**
Stool	Geographic location	NA	NA	NA	NA	NA	NA	**<0.001**	NA	NA	NA	NA	NA	NA	0.789
Stool, urine, stone	Sample type	NA	NA	NA	NA	**<0.001**	0.0923	NA	NA	NA	NA	NA	0.61	0.617	**<0.001**
Urine	Sex	NA	NA	NA	NA	**0.0205**	0.434	0.49	NA	NA	NA	NA	0.198	0.134	0.395
Urine	Geographic location	NA	NA	NA	NA	NA	NA	0.299	NA	NA	NA	NA	NA	NA	**0.052**
															
Beta-diversity															
Stool	12m abx	NA	NA	NA	NA	**0.054**	NA	NA	NA	NA	NA	NA	**0.042**	NA	NA
Stool	Age group	0.289	NA	NA	1	0.504	NA	**0.001**	**0.027**	NA	NA	0.949	0.167	NA	**0.001**
Stool	USD status	**0.002**	0.274	**0.026**	0.351	0.72	NA	**0.019**	**0.001**	0.059	**0.023**	0.227	0.923	NA	0.229
Stool	USD status × age group	0.323	NA	NA	0.254	0.831	NA	**0.001**	0.245	NA	NA	0.204	0.357	NA	**0.011**
Stool	USD status × sex	NA	NA	NA	**0.011**	**0.018**	NA	0.227	NA	NA	NA	**0.049**	0.737	NA	0.564
Stool	USD status × geographic location	NA	NA	NA	NA	NA	NA	**0.001**	NA	NA	NA	NA	NA	NA	**0.001**
Stool	Geographic location	NA	NA	NA	NA	NA	NA	**0.001**	NA	NA	NA	NA	NA	NA	**0.049**
Stool, urine, stone	Sample type	NA	NA	NA	NA	**0.001**	**0.001**	**0.001**	NA	NA	NA	NA	**0.001**	**0.001**	**0.001**
Urine	30d abx	NA	NA	NA	NA	**0.025**	NA	NA	NA	NA	NA	NA	**0.032**	NA	NA
Urine	Age group	NA	NA	NA	NA	**0.001**	0.773	**0.001**	NA	NA	NA	NA	**0.006**	0.867	**0.003**
Urine	USD status	NA	NA	NA	NA	**0.03**	NA	NA	NA	NA	NA	NA	0.882	NA	NA
Urine	USD status × 12m abx	NA	NA	NA	NA	**0.034**	NA	NA	NA	NA	NA	NA	0.059	NA	NA
Urine	Hypertension	NA	NA	NA	NA	**0.004**	NA	NA	NA	NA	NA	NA	**0.201**	NA	NA
Urine	Sex	NA	NA	NA	NA	**0.001**	**0.002**	**0.001**	NA	NA	NA	NA	**0.001**	0.057	**0.001**
Urine	Geographic location	NA	NA	NA	NA	NA	NA	**0.001**	NA	NA	NA	NA	NA	NA	**0.001**
Urine	Water intake	NA	NA	NA	NA	**0.006**	NA	NA	NA	NA	NA	NA	**0.015**	NA	NA
Urine	WT group	NA	NA	NA	NA	**0.012**	NA	NA	NA	NA	NA	NA	**0.018**	NA	NA

a The table column subheadings indicate study references (hyperlinked). M-A, meta-analysis. Metadata categories are included if the results were significant for at least one study using either OTUs or ASVs. NA, metadata category was not collected for that study. Significant values are indicated in boldface. abx, antibiotics; WT, weight.

10.1128/mBio.02007-21.2 FIG S1 Significant alpha diversities from microbiome study meta-analysis by ASVs. (A) USD status and age group for stool. Age groups include <30 years old, 30 to 50 years old, 51 to 70 years old, and >70 years old. (B) USD status in stool samples from different study locations: Cleveland (USA), Nanning (China), Vancouver (Canada) and Sutarwadi (India). Download FIG S1, PDF file, 0.07 MB. Copyright © 2021 Kachroo et al. 2021 Kachroo et al. https://creativecommons.org/licenses/by/4.0/ This content is distributed under the terms of the Creative Commons Attribution 4.0 International license.

10.1128/mBio.02007-21.3 FIG S2 Significant beta diversities from microbiome study meta-analysis with ASVs. (A) USD status in stool samples from different study locations: Cleveland (USA), Nanning (China), Vancouver (Canada), and Sutarwadi (India). (B) USD status and age group for stool. Age groups include <30 years old, 30 to 50 years old, 51 to 70 years old, and >70 years old. (C) Sex for urine. (D) Age group for urine. Download FIG S2, PDF file, 0.3 MB. Copyright © 2021 Kachroo et al. 2021 Kachroo et al. https://creativecommons.org/licenses/by/4.0/ This content is distributed under the terms of the Creative Commons Attribution 4.0 International license.

**FIG 4:**
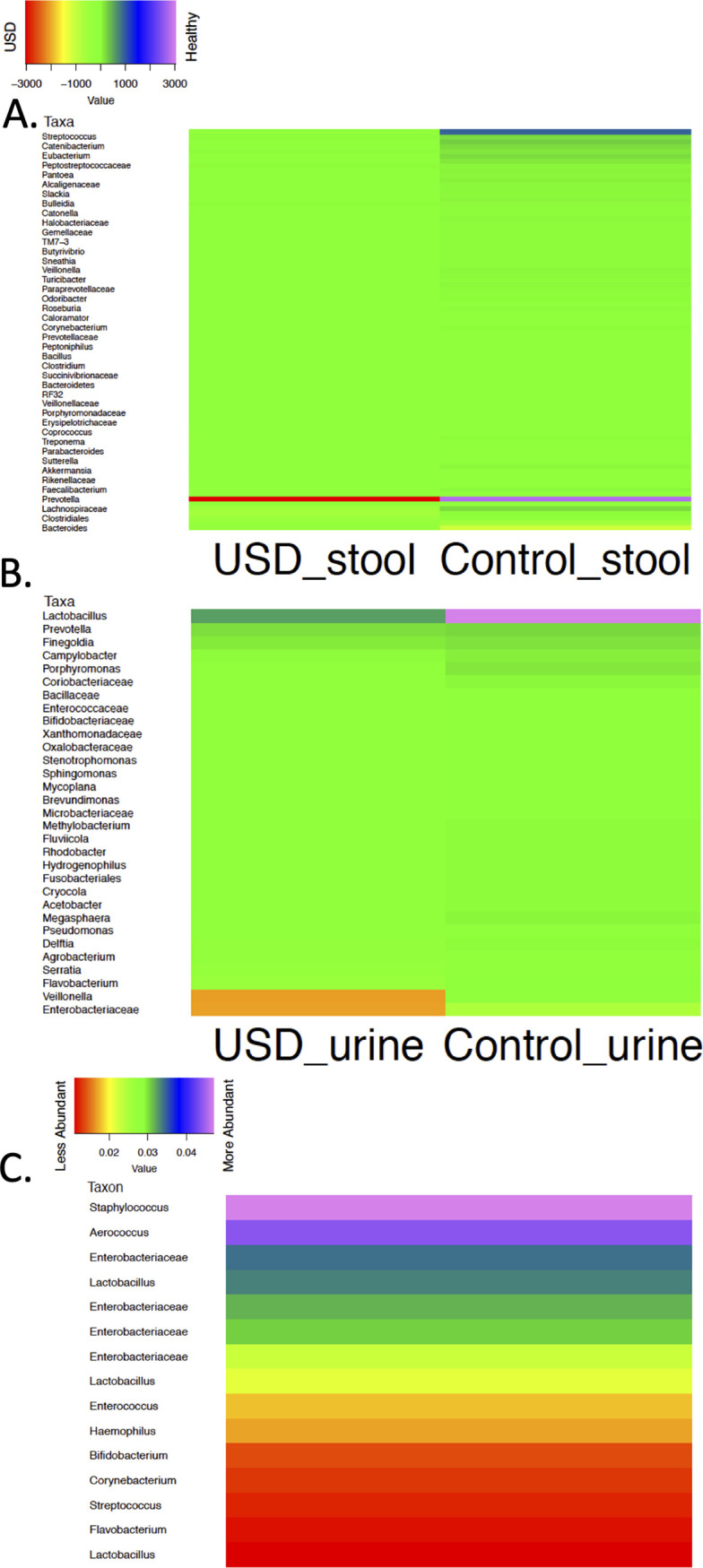
Heatmaps showing the most common dysbiotic taxa based on OTUs by sample grouping and sample type. The taxa were identified as pathogenic to beneficial (A and B) and from less abundant to most abundant (C). (A) Comparison by USD status in stool. Differential abundance analysis showed different *Prevotella* OTUs as the most healthy- and USD-associated taxa. (B) Comparison by USD status in urine. *Lactobacillus* was the most healthy-associated in urine, with the *Veillonella* and *Enterobacteriaceae* most associated with USD. (C) Bacterial stone analysis. Across two studies, OTUs from the Staphylococcus and *Aerococcus* genera dominated the microbiome, with several *Enterobacteriaceae* present at high abundance.

Differential abundance analysis based on OTU classification revealed that different OTUs within the genus *Prevotella* were the most frequently associated in the gut of both healthy and USD individuals (Fig. 4A). In the urinary tract, the genus *Lactobacillus* was most associated with healthy individuals, while the family *Enterobacteriaceae* and genus *Veillonella* were the most associated with USD (Fig. 4B). In stone samples, across two studies, specific OTUs from the Staphylococcus and *Aerococcus* genera dominated the microbiome, with several *Enterobacteriaceae* present at high abundance (Fig. 4C). With ASV assignment, differential abundance analysis showed that in the stool, *Lachnospiraceae* were the most associated with the healthy group and *Bacteroidaceae* were most associated with the USD group. In the urinary microbiome, *Veillonellaceae* were associated with the most healthy subjects, and the *Actinomycetaceae* and *Enterobacteriaceae* were most associated with USD. Finally, analysis of the stone microbiome revealed the stone microbiota to be dominated by the *Enterococcus* (see Fig. S3). Interestingly, *Oxalobacter*, which is the most researched bacterial genus relating to USD (4), was not significantly enriched in the gut of control subjects from any study.

10.1128/mBio.02007-21.4 FIG S3 Heatmaps showing the most common dysbiotic taxa based on ASVs by sample grouping and sample type. The taxa were identified as pathogenic to beneficial (A and B) and from less abundant to most abundant (C). (A) Comparison by USD status in stool. Differential abundance analysis showed Lachnospiraceae as being the most associated with the healthy group and the Bacteroidaceae most associated with the USD group. (B) Comparison by USD status in urine. *Veillonellaceae* were the most healthy-associated in urine, with the *Actinomycetaceae* and *Enterobacteriaceae* were most associated with USD. (C) Bacterial stone analysis reveals the stone microbiota to be dominated by the *Enterococcus*. Download FIG S3, PDF file, 0.1 MB. Copyright © 2021 Kachroo et al. 2021 Kachroo et al. https://creativecommons.org/licenses/by/4.0/ This content is distributed under the terms of the Creative Commons Attribution 4.0 International license.

## DISCUSSION

Microbiome-wide association studies are in their infancy for urolithiasis, with a dozen studies published since 2016 (5–16). In the current meta-analysis of MWAS studies, we found strong associations between the gut microbiome and incidence of USD (Fig. 1 and 2 and Table 2). Furthermore, we found that the microbiome exhibited significant associations with stone composition, age group, and study location, in one-way (all three variables) and two-way (age group and study location) analyses with incidence of USD (Fig. 1 and 2; Fig S1 and S2; Table 2). However, these results were dependent on the bioinformatic classification scheme used and were limited by sampling sites, number of data sets made available from published studies, along with the level of detail and accuracy of the clinical metadata collected. Therefore, the significance of the results must be understood within the context of the study limitations.

A primary limitation of the current meta-analysis was that many published MWAS did not make their raw data available. We note that NIH guidelines state that all raw genome or metagenome data and metadata should be submitted to the sequence read archive no later than 45 days after quality control, which is also a requirement by most scientific journals prior to publication. In addition to making data available, to push MWAS toward greater clinical relevance, field-specific standardization of methods and analyses must be achieved. The American Urological Association, for instance, has a published set of guidelines for the medical management of kidney stones that include a number of standardized practices such as a screening evaluation of blood and urine chemistries, stone composition analysis, quantification of stone burden, among other considerations (19). Clinical MWAS should be held to the same level of consistency.

While there were significant associations between the microbiome composition and study location, both independent and dependent of USD status (Fig. 1 and 2; Fig. S1 and S2; Table 2), data from each study location were generated by different laboratories with variable protocols for sample collection/storage, DNA extraction, and sequencing. It is well accepted that the experimental approach in metagenomic studies can have a big effect on the downstream data and interpretation (20). Thus, our meta-analysis is inconclusive when it comes to an association between the microbiome and study location and again bolsters the rationale for field-specific standardization of experimental approaches to the greatest extent possible. Despite the differences by study location, we also found a significant association between the microbiome composition and age group, both independent and dependent of USD status (Fig. 1 and 2; Fig. S1 and S2; Table 2). Age is a well-known risk factor for USD (21) and an independent modifier of the gut microbiota (22). Finally, stone composition exhibited a significant association with the urinary tract microbiome, but not the microbiome from the gut or the stones themselves (Fig. 1 and 2; Fig. S1 and S2; Table 2). These data may have important implications for the pathogenesis of stones. Specifically, the results suggest that the urinary tract microbiome, but not the gut microbiome, influences the host environment to indirectly promote or inhibit stone formation. The lack of association between stone type and the stone microbiome suggests that the bacteria do not play a direct role in lithogenesis. Other clinical metadata such as sex, body habitus, medications, diet, comorbidities, water intake, family history of USD, and other known risk factors for USD were only available for a single study. Thus, we cannot make any conclusions for these environmental factors in the current meta-analysis.

Finally, the meta-analysis results derived from either an OTU or ASV classification scheme were similar, differing only by whether USD status by itself had a significant association with microbiome composition. In our meta-analysis, we found that OTU classification produced greater within and between group discriminatory power, particularly for metadata where we expect to find clear differences such as sample type (stool, urine, and stone) and the urinary tract of males and females (Fig. 3). Thus, while some studies have found that ASV’s discriminate between ecological patterns more effectively than OTUs (23), we found the opposite trend here. The differences between OTU and ASV classification strategies can lead to differences in microbial profiles (18). However, with stringent postclassification quality control measures, the microbial profiles based on OTUs and ASVs are similar, as was the case for our meta-analysis. Given the results of our meta-analysis, it remains to be seen which classification strategy more accurately represents the diversity and taxonomy seen in microbiome samples as few studies have conducted direct comparisons of the two strategies using real world data. Another aspect that must be considered, which applies to both ASV and OTU strategies, is that both living and dead cells are sequenced to generate microbial profiles. However, sequencing data and enhanced culture data are generally concordant indicating that sequenced microbes provide an accurate representation of the living microbiome (16, 24).

Differences between patients and controls, based on OTU classification, were primarily driven by the genera *Prevotella* and *Lactobacillus* in the gut and urinary tract, respectively, to be strongly associated with healthy individuals, while bacteria from the family *Enterobacteriaceae* were strongly associated with USD in the urinary tract (Fig. 4). While the *Enterobacteriaceae* were also the most abundant taxa in the stone microbiota, specific OTUs belonging to the Staphylococcus and *Aerococcus* genera were more abundant than any one *Enterobacteriaceae* OTU. Interestingly, other strains of *Prevotella* were also strongly associated with the gut microbiota of USD patients. These data indicate that some strains of *Prevotella* may have anti-lithogenic activities whereas others produce prolithogenic activities. Since the *Prevotella* are among the most abundant genera in the gut, it will be important to delineate the roles of specific strains as it pertains to USD. Importantly, our data suggest that the urinary tract microbiome plays a greater role in pathogenesis than either the gut or stone microbiota, as indicated by the stone composition results, which can help to focus future mechanistic studies.

We performed the most comprehensive meta-analysis of publicly available data from clinical MWAS microbiome studies of USD to date. The results show that despite the limitations of the meta-analysis, there was a significant association between microbiome composition and USD status, which provides strong evidence for a role of the human microbiota in the pathogenesis of USD. In addition, there is evidence for stone composition, age, and study location as factors that influence the gut and urinary microbiome in ways that impact USD pathogenesis. However, additional data sets that consistently define these metrics would improve confidence in those results. Overall, our results provide a strong rationale for the field-specific standardization of experimental protocols, inclusion of all potentially pertinent anatomical niches, and greater collection and reporting of clinical metadata to ensure meaningful questions can be appropriately addressed.

## MATERIALS AND METHODS

### Study selection.

A comprehensive literature search of Google scholar, Scopus, and PubMed using the keywords “microbiome” AND “urolithiasis” OR “urinary stone disease” OR “nephrolithiasis” was performed to identify relevant clinical microbiome studies associated with USD published prior to April 2021 for inclusion. We included studies that focused either on the gut microbiota, the urinary microbiota, or both. These distinct microbiomes both potentially contribute to USD, and it is important to delineate which influences the onset of USD most, which can only be done through comparative analyses. Eligible studies were required to meet the following inclusion criteria: all studies had to (i) be solely focused on assessing the relationships between the microbiome and USD; (ii) use human clinical samples; (iii) include a comparative “control” non-USD cohort, unless the study was solely focused on the microbiota of stones; (iv) perform 16S ribosomal RNA (rRNA) gene sequencing; (v) have publicly available raw data and applicable metadata freely available for download or made available upon request; and (vi) be written in English.

Studies were excluded if they were review articles, editorials, or conference abstracts without full data available. Corresponding authors of studies that met all other eligibility but without publicly available raw data were also contacted directly via email to provide the required data in order to be included in the analysis. Since this study utilized publicly available anonymous data from known publications, institutional review board approval or patient consent were not required.

### Data analytical process.

Raw data for the meta-analysis was downloaded from the respective sequence read archive (SRA) accession numbers SRP140641, SRP140933, SRP066940, SRP103884, SRP125171, and SRP125191 for data analysis. Data from each study were downloaded, quality controlled, and trimmed in DADA2 (23). Using the Silva 138 SSURef and NCBI databases (25) as reference databases for mapping, sequences were assigned to either OTUs or ASVs in Qiime (26) or DADA2 (23), respectively. Chimeras, as well as taxa classified as eukaryotes, mitochondria, or chloroplasts, were removed from further analysis. Data sets were normalized with a DESeq2 normalization protocol which corrected for sequencing depth and composition bias across samples (27).

Alpha- and beta-diversities were calculated with the phylogenetic metrics, PD_whole_tree, and weighted UniFrac distance matrices, both within and between studies (28, 29). For analysis, several clinical metadata categories were examined based on clinical records unless otherwise noted. These included USD status (an active episode of USD or no history of the disease), age group (<30 years old, 30 to 50 years old, 51 to 70 years old, and >70 years old), city of study location, sex, weight group (<70 kg, 70 to 90 kg, 91 to 110 kg, 111 to 130 kg, and >130 kg), stone composition (defined for stones with >65% of a single mineral), antibiotic use in the 30 days or 12 months prior to sampling (all classes), gout, diabetes, hypertension, diet (self-reported as omnivore, pescatarian, vegetarian, Mediterranean, or low carbohydrate, in addition to number of servings/week of meat, desserts, fruits, veggies, and bread), and water intake (based on self-reported 8-oz. glasses of water/day). For alpha- and beta-diversity analyses, all two-way comparisons were made between USD status and other metadata categories both within and between studies.

Differential abundance of taxa between individuals with USD and controls with no history of USD was assessed using the DESeq2 algorithm (27). To determine the most dysregulated taxa in the gut and urinary tract between USD patients and controls, significantly different ASVs/OTUs were reduced to the lowest assigned taxonomy. The number of significantly different ASVs/OTUs assigned to those taxa were normalized to the total number of ASVs/OTUs in those taxa for the whole data set. The values normalized to taxon diversity were ranked as more healthy-associated for those taxa with higher values in the controls or more USD-associated for those taxa higher values in USD patients. For the stone microbiome, which is less diverse and does not have a control population, taxa were determined by ranking each ASV/OTU by the mean relative abundance across all samples in the data set. Within each study, *P* values were the false discovery rate corrected for multiple comparisons when applicable. Study-based heterogeneity in the results was assessed by calculating the I^2^ value of the alpha- and beta-diversity metrics in the metamicrobiomeR package for R statistical software (30). The analytical code is found at https://github.com/amill017/USD_metaanalysis_2020. Metadata are provided as Text S1 in the supplemental material.

10.1128/mBio.02007-21.1 TEXT S1 Metadata used for analyses. Download Text S1, TXT file, 0.3 MB. Copyright © 2021 Kachroo et al. 2021 Kachroo et al. https://creativecommons.org/licenses/by/4.0/ This content is distributed under the terms of the Creative Commons Attribution 4.0 International license.

### Data availability.

Raw data for the meta-analysis was downloaded from the respective sequence read archive (SRA) accession numbers SRP140641, SRP140933, SRP066940, SRP103884, SRP125171, and SRP125191 for data analysis. Scripts used for analysis can be found at https://github.com/amill017/USD_metaanalysis_2020. The metadata used for analyses is provided as in Text S1 in the supplemental material.
