# Supplementary figures and images for: Meta-analysis of Clinical Microbiome Studies in Urolithiasis Reveal Age, Stone Composition, and Study Location as the Predominant Factors in Urolithiasis-Associated Microbiome Composition
Source: mBio. 2021 Aug 10;12(4):e02007-21. doi: 10.1128/mBio.02007-21 (PMC8406293; doi:10.1128/mBio.02007-21)

**A. Stool x USD-status x Age\_group**

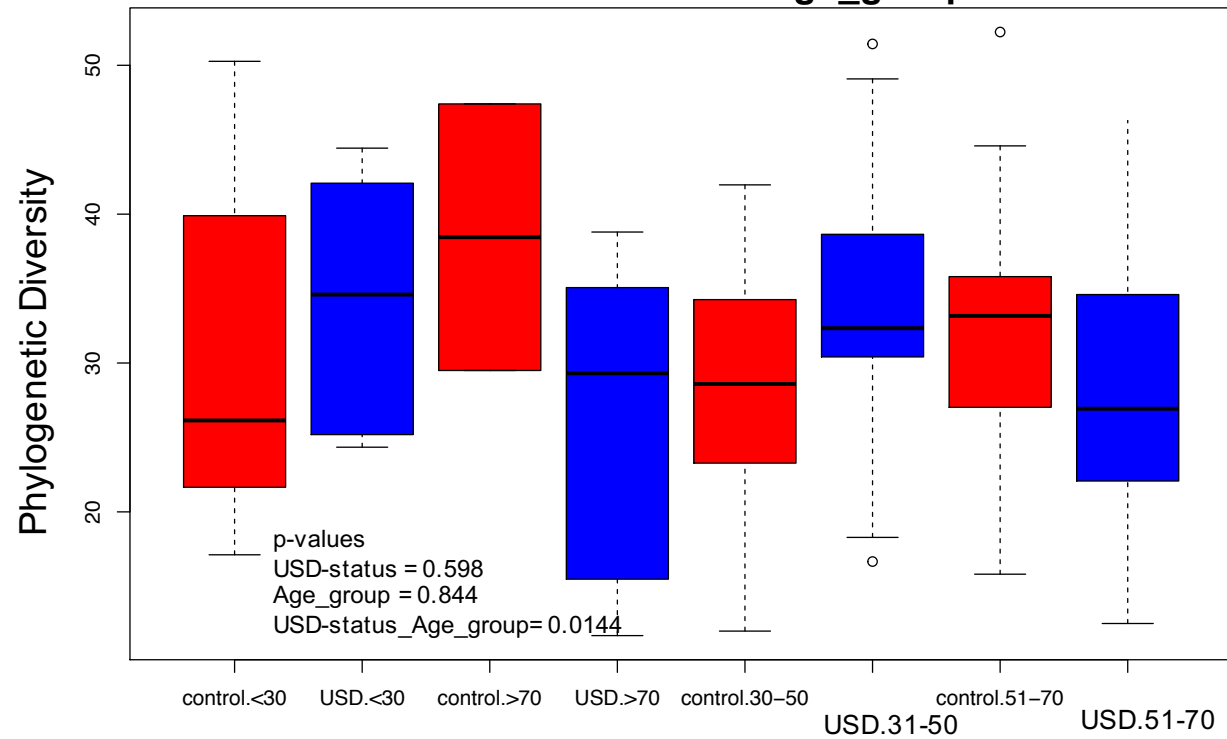

**B. Stool x USD-status x Study location**

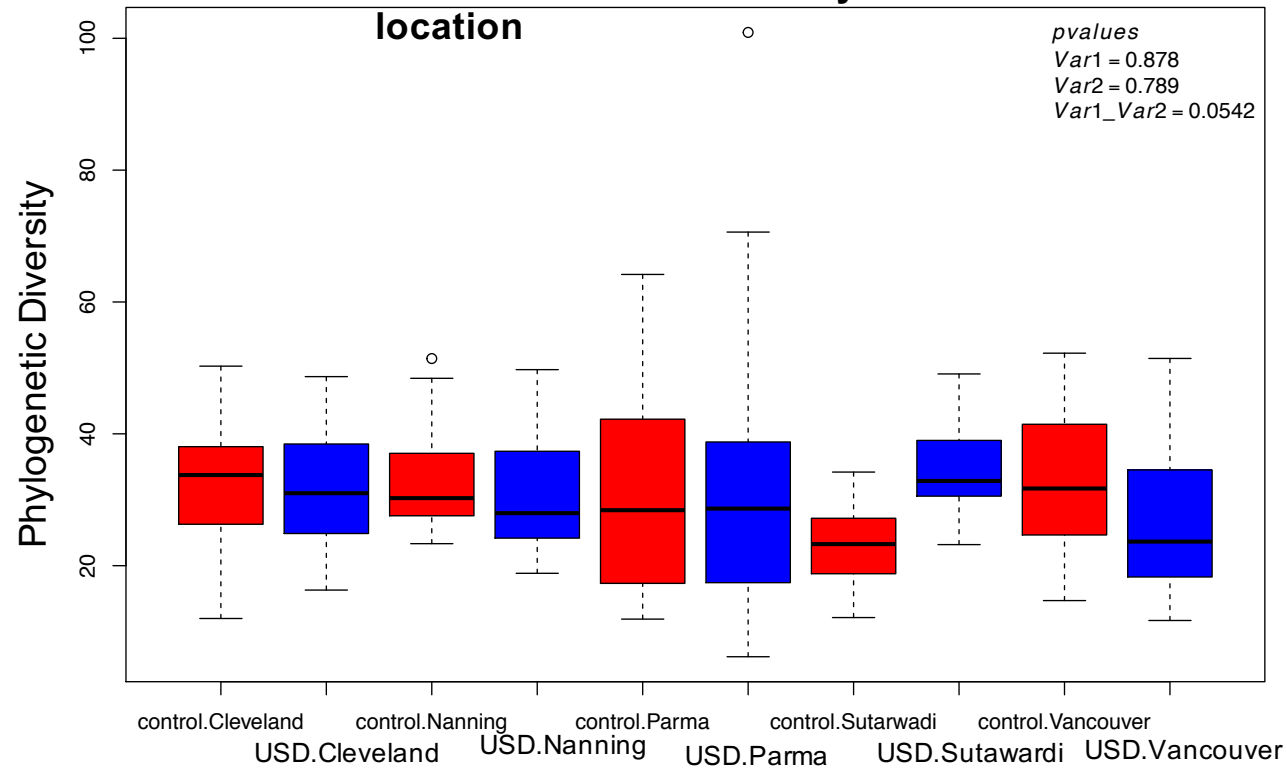

Supplement: FIG S1 [file mbio.02007-21-sf001.pdf]

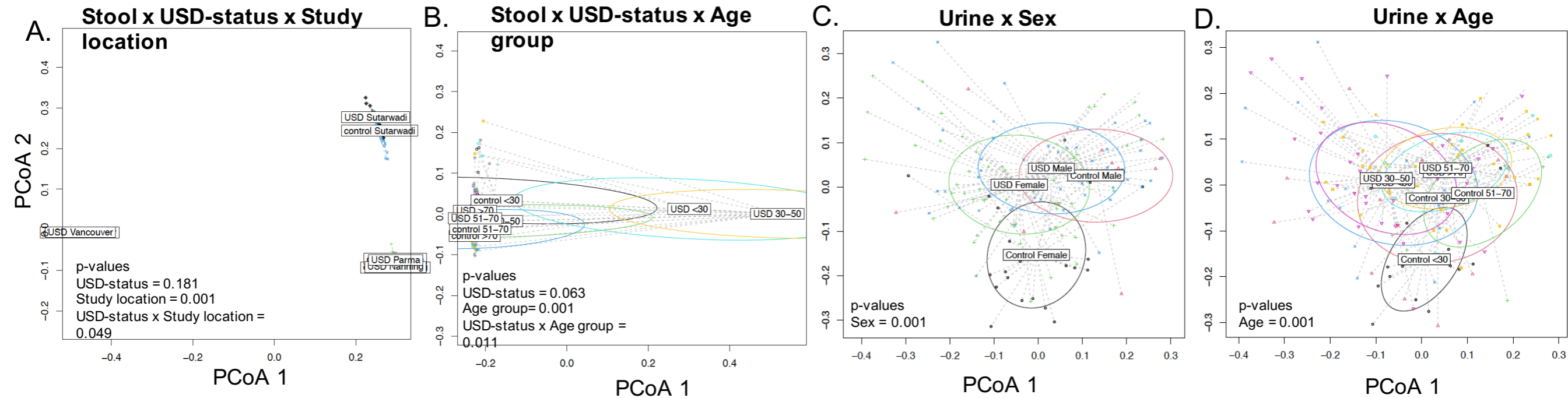

Supplement: FIG S2 [file mbio.02007-21-sf002.pdf]

A.

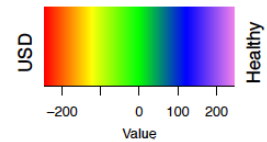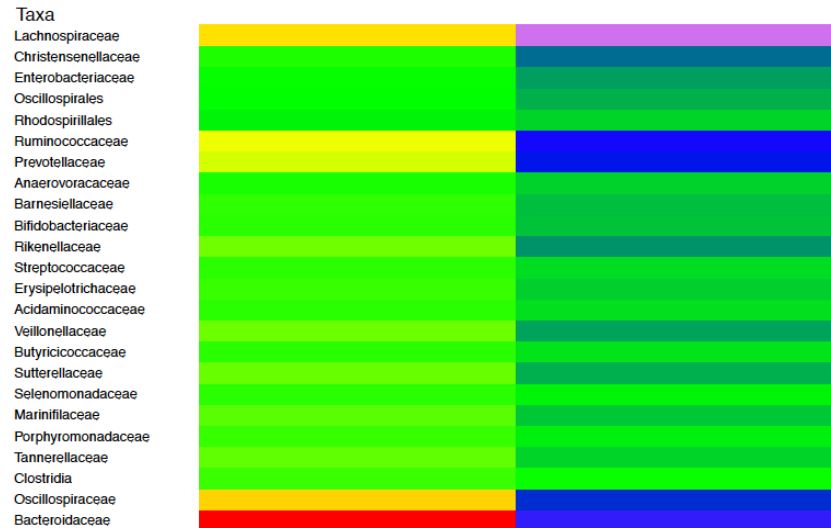

USD\_stool Control\_stool

B.

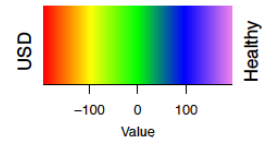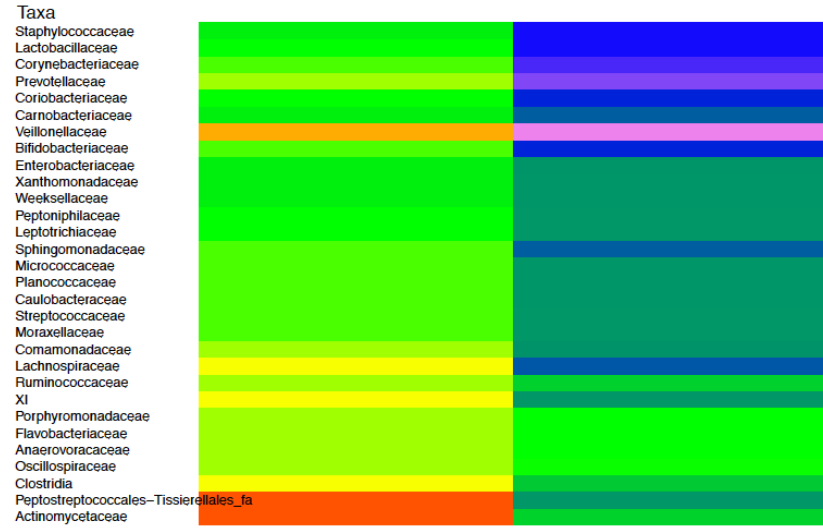

USD\_urine Control\_urine

C.

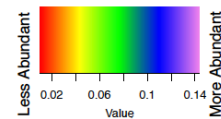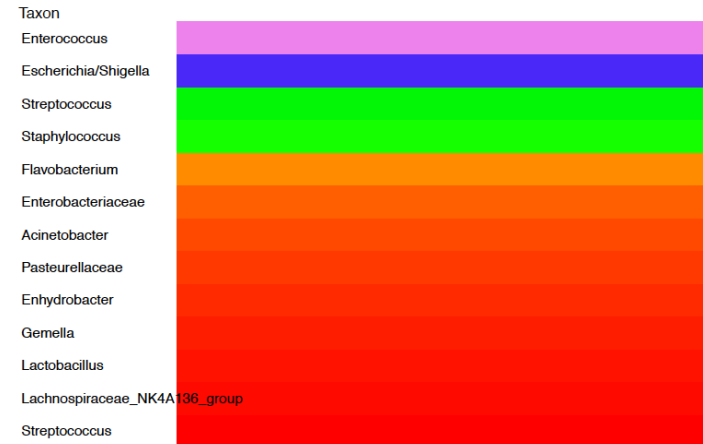

Stone  
Microbiota

Supplement: FIG S3 [file mbio.02007-21-sf003.pdf]
